# Assessing the Feasibility of a Multicenter Transition Intervention Model Across Adolescent Secure Services in England (MOVING FORWARD): Protocol for a Feasibility Cluster Randomized Controlled Trial

**DOI:** 10.2196/29273

**Published:** 2021-10-22

**Authors:** Maria Livanou, Rebecca Lane

**Affiliations:** 1 Department of Psychology School of Law, Social and Behavioural Sciences Kingston University Kingston Upon Thames United Kingdom

**Keywords:** transition, intervention, young people, feasibility cluster randomized trial, cluster randomized controlled trial, secure hospitals, outcomes, adolescents, patients

## Abstract

**Background:**

Young people moving from adolescent secure inpatient units to adult care in the United Kingdom have multiple and complex needs and are more likely to experience poor transition outcomes. Poorly managed transitions can lead to enduring use and dependency on mental health services. However, there is a lack of knowledge about the feasibility of transitional care models.

**Objective:**

This paper presents the protocol for a study that aims to test a feasibility cluster randomized controlled trial for young people transitioning from adolescent secure services to adult-oriented settings. The overarching aim of the MOVING FORWARD study is to provide a preliminary estimate of the effectiveness and cost-effectiveness of a new transition intervention model and to inform a future full-scale cluster randomized controlled trial.

**Methods:**

The design of the study is a 3-arm feasibility cluster randomized controlled trial comparing the MOVING FORWARD intervention against standard transition preparation conducted at 6 adolescent secure services, of which 4 units will receive the intervention and 2 will serve as controls. Eligible young people between 17-19 years, their parents/carers, and key workers will be invited to participate. Young people and parents/carers will be allocated to two conditions (young people alone and young people with a parent/carer) and will receive 4 transition preparation workshops across 6 months. Six adolescent secure hospitals will be randomly allocated, stratified by area and service type. Data will be collected at 3 time points: baseline (T0), 6-12 months postintervention (T1), and 18-24 months postbaseline (T2). Primary and secondary outcomes will be based on assessment measures and interviews conducted at T1 and T2.

**Results:**

A total of 13 young people and 17 staff members have contributed to the intervention design through online advisory groups on the design of the study and important themes for transition. We have also consulted members of the public (a steering group) including 2 young people who have transitioned to the community and 2 parents/carers. Common identified themes included appropriateness of module content and support during delayed transitions. The content of the intervention will be finalized during the first 6 months of the study. Participants will be recruited over the course of 6 months. An intraclass correlation coefficient will be calculated to inform the power of the sample size for a further large-scale trial. With a sample size of 50, we will be able to estimate a dropout rate of 80% (95% CI –11% to 11%).

**Conclusions:**

This research will provide practitioners and policy makers with an evidence-based framework of how training and familiarization with the prospective transitions can yield positive outcomes. This study will test whether a psychosocial intervention can be implemented in adolescent secure hospitals. The results will identify barriers and facilitators to the proposed intervention and will enable services to reflect on the quality of transitional care delivery.

**International Registered Report Identifier (IRRID):**

PRR1-10.2196/29273

## Introduction

### Background

Previous research has classified transitions as a global priority for chronic conditions [1]. There is substantial evidence that transitions from pediatric services to adult care in the United Kingdom are linked to the exacerbation of medical and mental health symptoms and poor life opportunities [2]. Young people moving from inpatient (tier 4) child and adolescent mental health services to adult-centered settings are more likely to experience poorly planned transitions due to inconsistent care and low transition readiness [3]. Fundamental differences in treatment approaches and care priorities have created a substantial gap between child and adult services and, coupled with low transitional readiness, have been found to be major barriers to positive transition outcomes [4]. Transitional care is particularly poor for young people in adolescent secure services, most of whom are detained under the jurisdiction of the Mental Health Act due to offending, and present with multiple comorbidities (including emerging personality disorders, neurodevelopmental and learning disabilities, and psychosis), for which many already experience prolonged stays in hospital [5]. As of 2016, there were 1283 young people in secure hospitals across England; approximately 43% of this cohort did not have a placement address when transitioning to the community [5]. Adolescents in these services, including medium and low secure units and psychiatric intensive care units (PICUs), constitute a neglected research and clinical group despite their multitude of needs and disadvantage (social adversity, trauma, high risk, and vulnerability).

Young people in contact with the youth justice system are more likely to experience multiple transitions across a range of services, including secure establishments to secure inpatient hospitals [6]. Six percent of young people in secure care have been in 10 or more placements before moving to inpatient secure hospitals [5]. Each setting follows different principles in their care approach and delivers different treatment models to the young people they accommodate. These constant changes in principles and care are particularly harmful and challenging for young people with learning disabilities, autism [6], and other neurodevelopmental disorders.

### The Need for a New Transition Model

There remains a dearth of research aiming to develop, implement, and evaluate transitional care models [6]. Effective and supportive transitions foster independence in young people and facilitate confidence in their treatment. The National Institute for Health and Care Excellence (NICE) 2016 guidelines explicitly state that self-efficacy (managing one’s own health) is a key priority for service commissioners and policy makers, and recommend evidence-based research in the form of cluster randomized controlled trials (cRCTs) in self-management training to improve transition preparation for special groups [7]. This study aims to address this gap and improve current processes for young people transitioning from adolescent secure services to adult-oriented care by (1) developing a psychosocial transition intervention with a developmental focus and (2) assessing the feasibility of undertaking a future trial of its clinical and cost-effectiveness.

Fragmented support during transitions can lead to enduring use and dependency on mental health services pertinent to long-term financial burden, limited employment opportunities and social functioning [8], as well as high reoffending rates and reinstitutionalization [5]. As such, transitional care has become a national priority in the United Kingdom to minimize the risk of poor transition preparation and increase positive outcomes. Recently developed practice guidelines on care transitions from adolescent secure services to adult services stress the importance of graded and flexible transitions aiming for a needs-based care model, contrasting to the age-based approach currently in use [9]. However, most available interventions are in line with adult models of care and may fail to include developmental perspectives [5], and there are no interventions or guidance targeting transitional care in secure settings. In addition, there remains a lack of well-defined planning and knowledge about the feasibility of specific models of transitional care that can reduce the public health burden of emerging adults at risk. The implementation of systematic interventions during institutional and health care transitions is highly likely to be cost-effective considering the costs associated to enduring mental health issues and secure hospital admission. This research is timely and falls within the scope of the National Health Service (NHS) Five Year Forward View in implementing new models of care for young people within the capacity of “personalized care” and borne from coproduction, whereby individuals are empowered using a strength-based approach. The research also aligns with the NICE 2016 guidelines, the Care Act 2014, the National Institute for Health Research (NIHR) infrastructure, and the NIHR Applied Research Collaboration South London, aiming to improve health care services for young people.

### Evidence for the Proposed Intervention

The proposed developing intervention is based on previous effectively established and implemented intervention models, including cognitive behavior therapy (CBT), parent-child interaction therapy, attachment-based therapy, social skills training (SST), and psychoeducational elements, which target self-efficacy and management of mental health and conduct problems tailored around goal-oriented approaches. SST and CBT group-based interventions are particularly effective in young people with autism [10]. Elements from these well-established approaches will contribute to behavior change in young people in secure care via upskilling them and building a goal-oriented approach toward their discharge. For example, elements from attachment-based and trauma-informed models will be used to increase confidence among young people and facilitate the “letting go” process. There is compelling evidence that these therapeutic models are effective for young people with challenging behaviors and complex trauma histories like those in secure care [11-14]. The current intervention will contribute to self-efficacy upon discharge. Key transition workers will be trained to deliver this intervention considering they are key attachment figures for young people. The intervention will start 6 months before they turn 18 years of age to allow time to prepare and avoid abrupt transitions. These interventions are more likely to be brief and successfully delivered in clinical settings, as in the proposed feasibility trial [13]. Group-based interventions delivered to young people with neurodevelopmental needs and mental health problems have shown increased self-determination and social functioning [15-18].

### Aims and Objectives

The MOVING FORWARD study aims to implement a new transition intervention model for young people transitioning from adolescent secure services to adult-oriented settings and test the feasibility of a future cluster trial measuring its effectiveness. The primary objectives are (1) to finalize the development of the intervention; (2) to determine the feasibility of conducting a full cRCT in adolescent secure inpatient units; (3) to test the feasibility and acceptability of trial procedures and materials, including recruitment, randomization, allocation, assessment tools, response rates, adherence and follow-up across 6 sites; (4) to determine the appropriateness of the proposed outcome measures; (5) to describe treatment as usual (controlled condition) across all sites; and (6) to qualitatively explore the views and experiences of health care staff delivering the intervention.

## Methods

### Intervention Development

**Figure 1:**
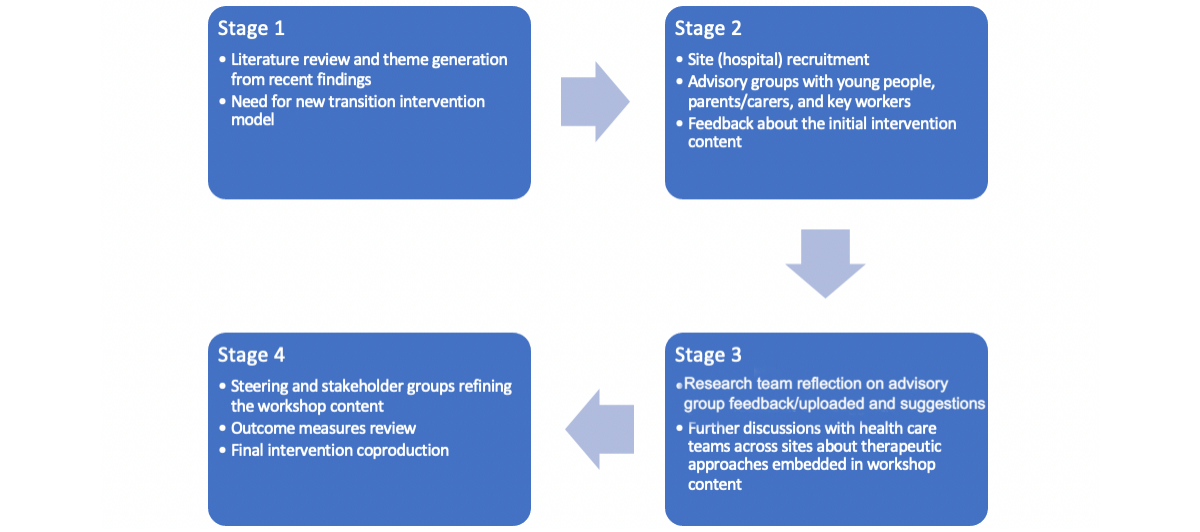
Development and refinement of the MOVING FORWARD intervention.

Figure 1 illustrates the coproduction plan and refinement process of the MOVING FORWARD intervention [19]. The intervention is in the process of development and will be finalized in the first 6 months of the study with the help of the steering and stakeholder groups, which were set up in the first and second stages of the coproduction phases. As of July 2021, the advisory groups have agreed on the overarching themes leading the 4 workshops: transition literacy; future plans; goal management; and expectations, attachment, and self-efficacy (Table 1). This research builds on an early career researcher grant awarded by Kingston University, which established advisory groups to coproduce a transition intervention for young people transitioning from adolescent secure services to adult services and their families. Advisory groups were set up with 13 young people with a previous, current, or upcoming transition experience from adolescent secure services to adult services, 2 parents of young people who had experienced this transition, and 17 staff from adolescent secure services. This study aims to implement this modular intervention and test the feasibility of a full clinical trial.

**Table 1:** Intervention themes and workshop content.

Intervention	Theme	Aims	Module tasks
Workshop 1	Transition literacy	Participants will be asked whether they understand the purpose of moving to adult services or an adult-oriented community and how they envision a positive transition. The elicited themes will inform the subsequent stages of the intervention	Role playing, vignettes/scenarios, videos, reflective discussion and writing, skills management
Workshop 2	Future plans	Participants will focus on education, employment, overall well-being, housing, and community living	Role playing, vignettes/scenarios, videos, reflective discussion and writing, skills management
Workshop 3	Goal management	Participants will be introduced to mental health symptom and risk management when in crisis, relapse and reintegration, support services, peer support, and role models and mentors	Role playing, vignettes/scenarios, videos, reflective discussion and writing, skills management
Workshop 4	Expectations, attachment, and self-efficacy	Participants will focus on what to expect in adult services, what difficulties they might encounter, what being an adult encompasses, what new responsibilities they will face depending on the type of placement they are admitted to, how the therapeutic style and alliance might change, parental involvement, hospital/community routine and structure, and older peers	Role playing, vignettes/scenarios, videos, reflective discussion and writing, skills management

### Feasibility Trial Design

This study is a mixed methods, multicenter, 3-arm, feasibility cRCT with a parallel design comparing the MOVING FORWARD intervention against standard transition preparation (usual care: Care Programme Approach [CPA] discharge and individual meetings with the responsible clinician) conducted at 6 adolescent secure sites, of which 4 units will receive the intervention and 2 will serve as controls. Young people across these adolescent secure services present with similar mental health and neurodevelopmental needs and complex trauma history. In this specific study, we will focus on those with emerging borderline personality disorder, psychosis, autism, learning disabilities, and conduct disorder. Data will be collected at 3 time points: baseline (time 0; T0), 6-12 months postintervention (time 1; T1), and 18-24 months postbaseline (time 2; T2). A cRCT will minimize the risk of contamination, which is high in the proposed study as elements from the intervention could be used for standard transition preparation.

A feasibility study will measure the acceptability of conducting a future, fully powered trial across different pools of young people in secure care and different settings across adolescent secure hospitals. This study will test how the future trial will look like, as well as the main research question and primary outcomes. Outcome measures and one-to-one semistructured interviews with young people, parents/carers, and health care staff will be conducted to explore the experiences of young people receiving the intervention and those who received treatment as usual (TAU). The practicality and integration of the intervention within the current system of care and use of resources will be explored with the participants. A total of 30 interviews will be sought to understand the mechanisms of impact.

### Training of Health Care Staff

A transition workshop will be delivered to a group of health care staff about preparing young people moving to adult services based on predetermined themes during the staff advisory group meetings (transition management, plans in action, coordination with receiving services, joint working, continuity of care, structural problems, infrastructural weaknesses, a structured health transition plan based on individualized needs, education in line with CPA reviews). This workshop will train health care staff to deliver the developing intervention to the young people who have agreed to participate in the study. Those delivering care as usual will not receive training. Health care staff will receive training 3 months pretransition in the form of two 45-minute workshops. The training will be standardized to increase intervention fidelity.

### Trial Conditions

#### Condition 1: T0 Baseline Pretransition (6-12 Months Before 18th Birthday)

As discussed with the advisory groups in the first stage of the coproduction/development phase, this intervention will include 4 modules delivered over 8 series of 2-day workshops. When the intervention is finalized, a manual will be developed including the goals and objectives of each workshop, skills developed, behavior expectations and management, themes that need to be covered, required materials for the sessions, and strategies for young people with neurodevelopmental needs. The content of the sessions will be the same across different groups of patients although adjustments will be applied to the intervention models based on each group’s mental health and developmental needs identified during the piloting phase with the lead sites (Ardenleigh and St Andrews). These adjustments will be discussed with the steering group to ensure that the intervention fidelity is not threatened. Additionally, monitoring procedures with the help of the research team across the study will enhance intervention fidelity. For example, young people with neurodevelopmental needs may need additional help in terms of mental health literacy and understanding the purpose of their prospective transition. The workshops will be delivered across 4 days, and there will be 4 sessions targeting different domains of transition. Staff members who attended the transition training will deliver these workshops as they are key attachment figures to the young people. Each workshop will take between 30-40 minutes. Key workers from the advisory groups suggested that the intervention should be informed by attachment-based therapy, CBT, SST, and psychoeducational elements, which target self-efficacy and management of mental health problems.

#### Condition 2: T0 Baseline Pretransition (Young People and Parents/Carers)

The same series of workshops will be delivered with involved parents/carers to examine whether parental involvement in the process yields different outcomes.

#### Condition 3: T0 Baseline Pretransition (TAU)

This group of young people will receive TAU (standard transition care through the CPA) depending on the service, which may or may not involve transition preparation.

#### Conditions 1-3: T1 (6-12 Months Posttransition)

Young people and parents/carers (condition 2) who received an intervention and those who received care as usual (condition 3) will be visited and interviewed at the receiving placement 6-12 months postdischarge. Outcome measures will be completed.

#### Conditions 1-3: T2 (18-24 Months: Endpoint)

Young people and parents/carers (condition 2) who received the interventions and those who received care (condition 3) as usual will be followed up depending on an old or new placement and will be interviewed about continuity of care, mental health, overall well-being, education, employment, and relapse. Outcome measures will be completed.

### Allocation Strategy

The adolescent secure service is the unit of randomization and will be randomly selected until 6 sites are recruited. Sites were eligible if they provided low or medium secure care, or they were classified as PICUs. Six adolescent secure hospitals will be randomly allocated into 1 of 3 arms in a 1:1:1 ratio, stratified by area (south versus north) and type (medium versus low versus PICUs) first. Then participants at each site will be allocated to the same treatment. A total of 4 secure units will be allocated to the intervention arms and 2 units allocated to the control arm. Allocation will be performed (computer-generated allocation) by an independent member of the team who will be blind to the identity of the unit. Considering the nature of the intervention, young people and health care staff will not be blinded. The statistician and all team members collecting follow-up data, apart from the principal researcher and fieldworkers, will remain blind to the allocation of the units to trial arms.

### Participants

#### Young People

A total of 50 participants will be recruited on a rolling basis over 6 months from the commencement of the study. Any young person meeting the inclusion criteria listed below irrespective of their gender, ethnicity, religion, education, and disability status will be eligible for inclusion:

- If aged ≥17 years and eligible for transition from an adolescent secure hospital, including those experiencing delayed transitions;
- If formally diagnosed with a mental disorder, and/or learning disability, and/or neurodevelopmental problem.

Any young person meeting the following criteria will be not eligible for inclusion:

- If presenting with moderate intellectual impairment with an IQ<65;
- If in an acute phase of severe mental illness;
- Cannot provide consent due to physical disabilities and/or language problems or any other neurodevelopmental deficits.

#### Parents/Carers

A total of 20 parents/carers will be recruited pre-, during, and posttransition. Family therapists, responsible clinicians, and social workers from participating adolescent secure services will facilitate this process. A random sample of parents/carers will be interviewed at T1 and T2.

#### Health Care Staff

A total of 30 multidisciplinary team members will be sought for recruitment during the different phases of the study. Key workers involved in the transition planning, such as responsible clinicians, psychologists, family therapists, occupational therapists, social workers, nurses, and health care support workers, will be recruited. A random sample of health care staff will be interviewed postintervention delivery at T1.

### Recruitment and Support

**Figure 2:**
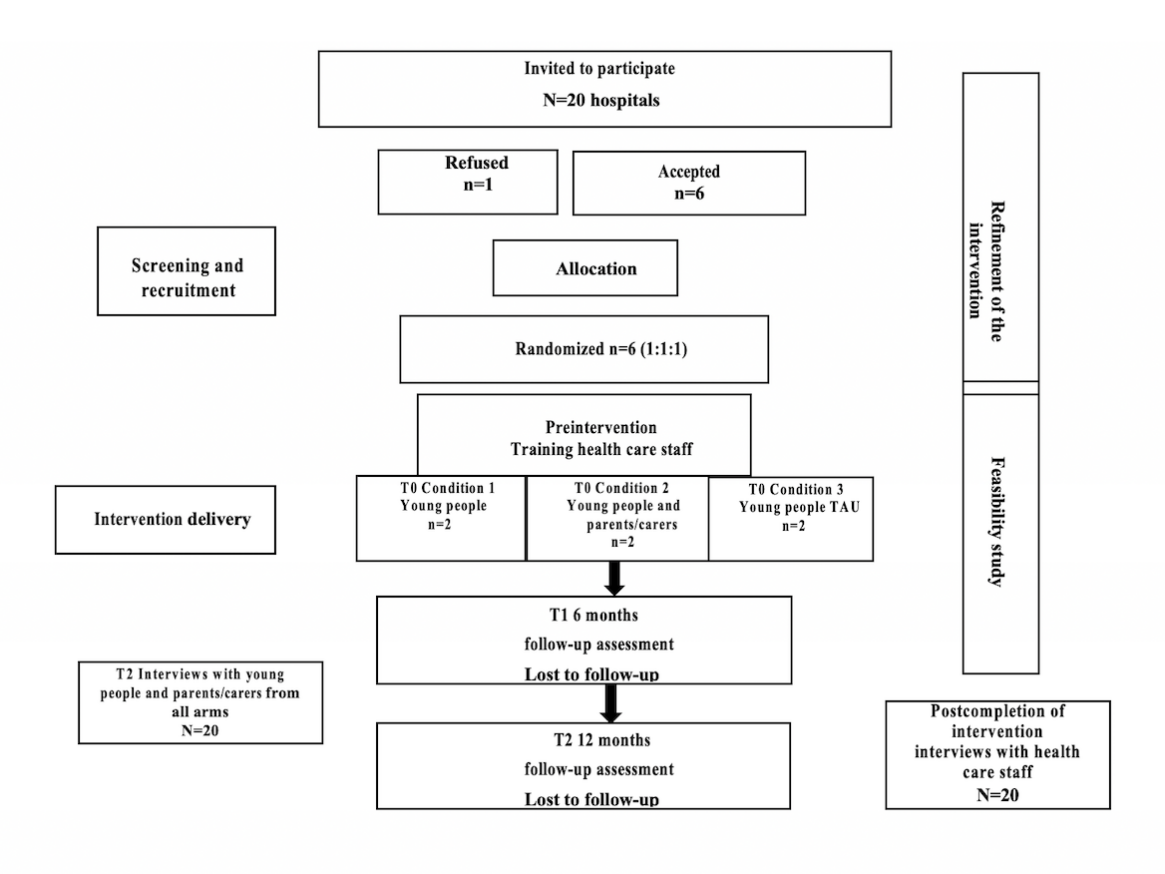
Diagram of the feasibility cluster randomized controlled trial. TAU: treatment as usual.

The research team will be informed by health care staff at each site about the young people’s eligibility and capacity to provide written informed consent. The local collaborator will introduce the study to all eligible young people with the help of a study leaflet, a visit or virtual meeting by the research team (to account for COVID-19), and posters advertised at each site. The information leaflets will consider young people’s developmental, language, and learning impairments. The “Bonding Plan,” which was adopted by the MILESTONE study (the largest European transition trial) will be used as a retention strategy with some minor adjustments depending on the setting. For young people in the community, gift vouchers will be provided. For those in secure hospitals, a voucher will be transferred to their account so that they can use it once they are released. Figure 2 presents more details.

### Sample Size

An intraclass correlation (ICC) coefficient will be calculated to inform the power of the sample size for a further large-scale trial. A statistician based at the joint Faculty of Health, Social Care and Education at Kingston University and St George’s University of London will be involved in the design of the feasibility cRCT. This feasibility study aims to estimate the required sample size for a definitive cRCT evaluation of the intervention. We will estimate the variability in the likely definitive trial outcome (improved mental health and adjustment to adult settings upon follow-up) using an ICC. The sample size of young people may vary between 40 to 60 (8 to 10 young people at each site) and parents/carers 20 to 30 (4 to 5 parents/carers at each site) as recommended [20]. With a sample size of 50, we will be able to estimate a dropout rate of 80% (95% CI –11% to 11%).

### Primary Outcome Measures for a Future Trial to Measure Improved Mental Health, Social Functioning, Adjustment, and Quality of Life

The suitability and acceptability of assessment tools will be based on feedback from the steering and stakeholder groups and key workers in secure hospitals a priori and ad hoc and response rates. Additional feedback will be sought from the participants after completion of the following questionnaires:

1. Transition Readiness Assessment Questionnaire (TRAQ) [21] (T0, T1, T2): to measure whether the transition intervention outcomes are linked to transition readiness predischarge;
2. Transition Related Outcome Measure (TROM) [22] questionnaire (T1, T2): to measure whether transition outcomes have improved as a result of the intervention;
3. Adult functioning assessed by the Specific Levels of Functioning Scale (SLOF) [23] (T0, T2): to measure social functioning and evaluate how the intervention has facilitated self-management and efficacy in young people;
4. Subjective well-being assessed by the Personal Adjustment and Role Skills Scale (PARS-III) [24] (T1, T2): to measure psychosocial adjustment–peer relations, hostility, risk in postdischarge, and if the intervention has facilitated the process of psychosocial reintegration;
5. Quality of life assessed by the World Health Organization Quality of Life Assessment (WHOQOL-BREF) [25] (T0, T1, T2): to determine if the intervention has improved quality of life—physical, psychological, and independence level—postdischarge;
6. EuroQol Five-Dimensional Questionnaire, Youth Version (EQ-5D-Y) [26] (T0, T1, T2): to determine if this cost-effectiveness tool is appropriate for a fully powered trial along with the intervention fidelity;
7. Data on preliminary resource use will be collected via semistructured interviews with health care staff, young people, and parents/carers. Service use schedules may be developed to assess cost-effectiveness.

### Analysis Plan

#### Health Economic Data and Analysis

All resources required to measure the intervention implementation including the time spent by staff in training and on delivering the intervention will be monitored with site visits and allocated local collaborators at each site. The EQ-5D-Y will be used to measure health-related quality of life and its sensitivity will be explored. Considering this is a feasibility study, collected data such as intervention and resource-associated costs will be collected with relevant questions via qualitative interviews (eg, clinical opinion) to inform the economic evaluation for the future full-scale trial.

#### Quantitative Analysis

Demographic characteristics will be summarized using descriptive statistics. The between-cluster and within-cluster variance will be determined. Sample sizes for a future definitive trial will be based on detecting a difference in outcomes (mental health and risk presentation) between trial arms, estimated using the TROM and based on combinations of different alpha (ie, significance level) and beta (ie, power) rates. The ICC will be calculated using the linear mixed model and will explore correlations between participants within the same cluster, as well as to inform sample size calculation for the definitive trial.

#### Qualitative Analysis

Recordings of interviews will be transcribed verbatim. Thematic analysis will be performed to identify key themes around transitions. The 6-step approach as suggested by Braun and Clarke [27] will be followed. Predetermined (derived from the literature) and data-driven codes will be used for transcription purposes. These codes will be refined to produce emergent themes. A constructionist approach will be followed to account for the impact of context and personal experiences. The transcripts will be examined for similarities, differences, and patterns. The experiences of young people will be explored to understand the intervention acceptability, implementation, and mechanisms of impact.

### Ethics

Approval by the Health Research Authority will be sought for the purposes of this research. The research team has extensive experience with submitting previous applications using the Integrated Research Application System to obtain ethical approval, including for vulnerable groups such as young offenders. Then, each site should provide an honorary contract or letter of access to the research team, facilitated by the Research and Development office at that site.

This protocol was submitted in January 2021 to the NIHR Advanced Fellowship Programme and has been internally and externally peer-reviewed by academic colleagues based at King’s College London and Kingston University.

## Results

### Important Themes Based on Advisory Group Feedback

The advisory groups highlighted themes of particular importance and relevance to the intervention, including communication and parallel care, transition delays, traumatic assessments, familiarity with adult services and key workers, and family engagement, and provided advice about the outcome measures. Young people described transition to adult services as scary, and they were keen to participate in the prospective feasibility study. A young person’s recommendation on providing peer support to improve self-management in the community was incorporated in the intervention. These areas have informed key aspects of the intervention, such as the inclusion of parents/carers, and its core modules, which improve the understanding and expectations of transitions and working with goals (described in more detail below and in Table 1).

### Timeline and Deliverables

The first year will be used for recruitment, finalizing the intervention, developing the protocol of the feasibility study, and training health care staff. During the second year, we will conduct follow-up assessments and data analysis. The final year will be used for report writing and dissemination of findings through patient and public involvement (PPI) and clinical collaborations to increase impact on policy transition guidelines and standard clinical practice. The deadlines outlined below will be followed strictly to manage the delivery of reports within the designated time frame. Study leaflets have been administered to potential participants already, and local collaborators have informed staff and young people about the feasibility study at all participating sites. Potential risks have been discussed with the advisory groups.

### Dissemination and Impact

Advisory group members, all of whom have direct or indirect experience of the transition from adolescent secure services to adult services, have expressed interest in providing PPI throughout the study to improve outcomes in design, data collection, analysis, and dissemination. One young person has been recruited as a member of the research team, and 2 parents and 2 young people have agreed to join the steering group, which will meet twice annually for 3 years. Additional PPI recruitment efforts are ongoing, aiming to recruit an additional young person and parent/carer to the steering group. A stakeholder group will be built including 1 NHS commissioner and 2 health care providers who have agreed to participate. The intervention will be finalized with the help of the steering and stakeholder groups, who will additionally review information sheets, consent forms, and outcome measures to check their appropriateness.

The findings are expected to be presented at national and international conferences, published in peer-reviewed journals, and communicated in regular study newsletters, meetings, and internal conferences at the participating sites. An internal networking event including adolescent and adult secure and community services will be organized to share the findings and receive input about the sustainability and inclusion of the proposed intervention in standard care. Kingston University in collaboration with the University of Warwick will hold a public dissemination event in which young people will reflect on their transition experiences. The findings will be also shared with NHS England and the Department of Health.

We will additionally work with young people who have returned to their communities, who will be supported to run local events in their catchment area to inform the public in accessible ways (using social media and YouTube) about transitioning to the community from secure care. Young people who have moved to adult secure hospitals will have an equal opportunity of being involved in the dissemination of the findings to their peers and their parents/carers by arranging internal community dissemination events in hospitals. A video will be created by young people who have moved to adult services to showcase to future cohorts.

### Progression Criteria and Feasibility Outcomes

A traffic light approach (stop-amend-go/red-amber-green) will be used and discussed a priori with PPI, advisory and local collaborators/key contacts, and research mentors about the following criteria:

- Recruitment: at least 80% of target
- Intervention fidelity: at least 80% of intervention workshops meet the fidelity criteria
- Intervention acceptability: at least 80% of staff can be trained and deliver the intervention (barriers and facilitators to intervention design)
- Follow-up numbers: at least 70% at T1 and 60% at T2
- Acceptability of assessment tools: at least 70% at T1 and 60% at T2

The outputs are as follows:

- Design a multicenter intervention for young people in transition;
- Design training manuals/toolkit for the health care staff involved in transition;
- Reflect on good practices across adolescent secure hospitals for transition to adult services and the community and management;
- Promote co-design and coproduction of evidence-based research.

The impact is as follows:

- Design of the future cRCT will promote effectiveness and cost-effectiveness;
- Assess barriers and facilitators to providing the proposed intervention;
- Help services to reflect on quality of care and performance pre- and postdischarge;
- Enable young people to voice their experiences and be dynamically involved in their care.

**Figure 3:**
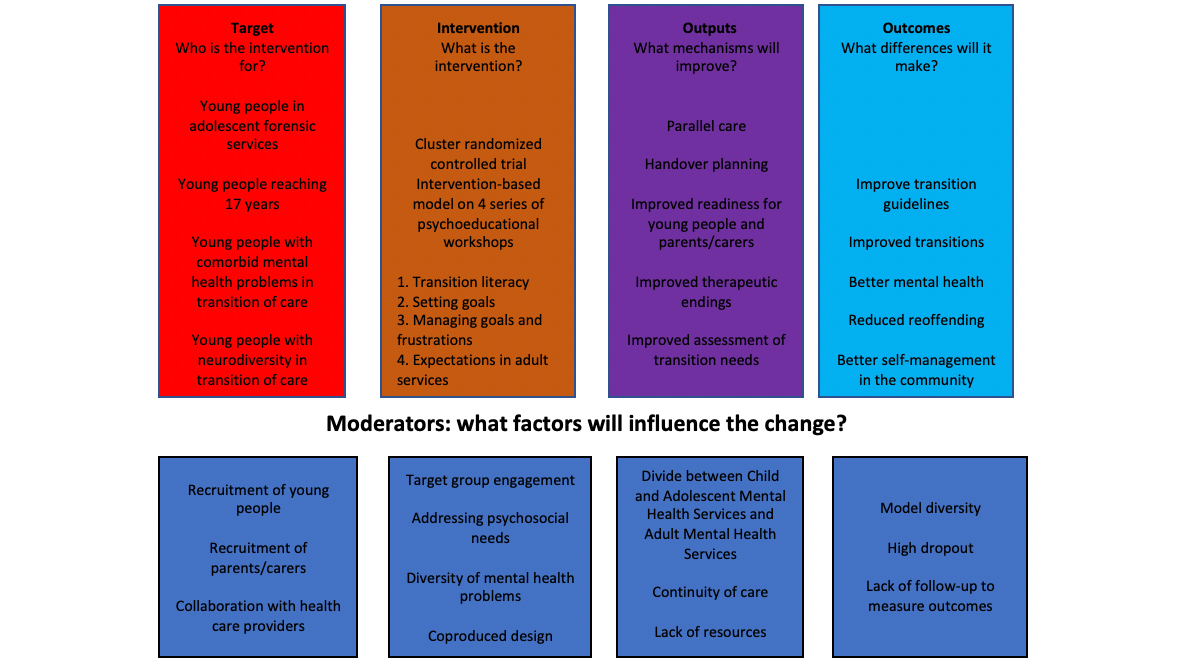
Logic model: outcomes for the full-scale trial.

Figure 3 presents the outcomes for the full-scale trial.

## Discussion

### Key Findings in Coproduction

This study aimed to describe the first and second stages of coproducing the MOVING FORWARD intervention. We collaborated with young people, parents/carers, and key workers from adolescent secure hospitals. In addition to addressing the gaps and concerns highlighted above, this research builds upon a previous NIHR Collaborations for Leadership in Applied Health Research and Care–funded PhD project titled *Transition of care in young offenders,* a multiphased study that illustrated the need for a national integrative care model that acknowledges the complexities involved in young people’s care journey. Factors found to impede effective transitions pertained to the severity of index offence, lack of parental involvement, disorganized service planning, and inconsistent clinical practice [20]. Groups at disadvantage were also highlighted, including young females with emerging borderline personality disorders and males with high-risk learning disabilities and long-term seclusion. Acknowledging the diversity in needs among young people has helped to coproduce and tailor the proposed intervention.

### Patient and Public Involvement

Young people and parents/carers are central to this study. Young people in secure care present with multiple comorbidities and challenging behavior, and do not have a dynamic role in decision-making as a result of being detained under the Mental Health Act legislation. Parents/carers are particularly concerned about transitions as they do not know what to expect and do not feel involved in the process. PPI will be sought at all stages of the study to improve outcomes in design, data collection, analysis, and dissemination. External PPI members will receive relevant training during the early stages of the study, and a point of contact from each site will be identified to ensure emotional support is provided in the event the young people feel distressed and direct them to appropriate services. A total of 5 young people and 5 parents/carers will be recruited to represent the study’s steering/PPI group and meet twice annually over the course of 4 years at St. George’s Hospital. One NHS commissioner and 2 health care providers will be involved in the steering groups.

Engagement of young people has been sought during the development of the intervention. The youth and parent/carer steering group will review information sheets, consent forms, and outcome measures to check their appropriateness. During the end of the study’s second and final year, young people from the advisory and steering groups will be asked to aid with the dissemination of the interim reports to their community and/or hospitals. The parent/carer advisory groups will be recruited from St Andrew’s and Cygnet’s Sheffield hospitals, and their views about the intervention model and how it can benefit young people will be collected. Feedback from all stakeholders will help to interpret the findings effectively and address how the intervention can fit future cohorts transitioning out adolescent secure services to facilitate the continuity of care.

## Abbreviations

CBT: cognitive behavior therapy
CPA: Care Programme Approach
cRCT: cluster randomized controlled trial
EQ-5D-Y: EuroQol Five-Dimensional Questionnaire, Youth Version
ICC: intraclass correlation
NHS: National Health Service
NICE: National Institute for Health and Care Excellence
NIHR: National Institute for Health Research
PARS-III: Personal Adjustment and Role Skills Scale
PICU: psychiatric intensive care unit
PPI: patient and public involvement
SLOF: Specific Levels of Functioning Scale
SST: social skills training
TAU: treatment as usual
TRAQ: Transition Readiness Assessment Questionnaire
TROM: Transition Related Outcome Measure
WHOQOL-BREF: World Health Organization Quality of Life Assessment
